# Competitive Protein Binding Assay of Naproxen by Human Serum Albumin Functionalized Silicon Dioxide Nanoparticles

**DOI:** 10.3390/molecules24142593

**Published:** 2019-07-17

**Authors:** Qian-Long Wang, Jing Xie, Jian Liang, Geng-Ting Dong, Li-Sheng Ding, Pei Luo, Lin-Sen Qing

**Affiliations:** 1 School of Pharmacy, Chengdu Medical College, Chengdu 610500, China; 2 Chengdu Institute of Biology, Chinese Academy of Sciences, Chengdu 610041, China; 3 State Key Laboratory for Quality Research in Chinese Medicines, Macau University of Science and Technology, Macau, China

**Keywords:** competitive protein binding assay, naproxen, horseradish peroxidase labeled naproxen

## Abstract

We have developed a new competitive protein binding assay (CPBA) based on human serum albumin functionalized silicon dioxide nanoparticles (nano-SiO_2_-HSA) that can be used for naproxen determination in urine. Compared with a conventional multi-well reaction plate, nano-SiO_2_ with a high surface-area-to-volume ratio could be introduced as a stationary phase, markedly improving the analytical performance. Nano-SiO_2_-HSA and horseradish peroxidase-labeled-naproxen (HRP-naproxen) were prepared for the present CPBA method. In this study, a direct competitive binding to nano-SiO_2_-HSAwas performed between the free naproxen in the sample and HRP-naproxen. Thus, the catalytic color reactions were investigated on an HRP/3,3′5,5′-tetramethylbenzidine (TMB)/H_2_O_2_ system by the HRP-naproxen/nano-SiO_2_-HSA composite for quantitative measurement via an ultraviolet spectrophotometer. A series of validation experiments indicated that our proposed methods can be applied satisfactorily to the determination of naproxen in urine samples. As a proof of principle, the newly developed nano-CPBA method for the quantification of naproxen in urine can be expected to have the advantages of low costs, fast speed, high accuracy, and relatively simple instrument requirements. Our method could be capable of expanding the analytical applications of nanomaterials and of determining other small-molecule compounds from various biological samples.

## 1. Introduction

Naproxen is a commonly used non-steroidal anti-inflammatory drug for the treatment of various acute and chronic pain, rheumatic and musculoskeletal disorders; it functions by specifically inhibiting cyclooxygenase 2 [1,2,3]. It is well known that overuse of naproxen is closely associated with a number of side effects including gastrointestinal damage, renal syndromes and hepatic insufficiency [4,5]. In particular, the efficacy, safety, and tolerability of naproxen were in accordance with the benefit and risk evaluation for the long-term administration of rheumatism patients. Thus, it is of high significance to develop a rapid, selective, and reliable approach to determine naproxen in biological samples.

There are a great many quantitative techniques that require precision instruments or manual operation that have been applied for naproxen determination, such as HPLC/UV [6], HPLC/MS [7], GC/MS [8], thin layer chromatography [9], capillary electrophoresis [10], spectrofluorometry [11], chemiluminescence [12], phosphorescence [13], potentiometric sensor [14], and flow injection analysis [15], coupled with a variety of sample pretreatment procedures, such as liquid-liquid extraction [7], solid-phase microextraction [16], ultrasound-assisted magnetic dispersive solid-phase microextraction [17], electrochemically controlled in-tube solid phase microextraction [18], functionalized multi-walled carbon nanotubes hollow fiber solid phase microextraction [19], carbon nanotube reinforced polyamide-based stir bar for sorptive extraction [20], molecularly imprinted polymer-coated magnetic multi-walled carbon nanotubes solid-phase extraction [21], water stable metal-organic framework packed microcolumn for online sorptive extraction [22], metal ion-mediated complex imprinted membrane for selective recognition [23], hyperbranched polyglycerol/graphene oxide nanocomposite reinforced hollow fiber solid/liquid phase microextraction [24], and imidazolium-based functional monomers imprinting [25]. However, these methods have two notable disadvantages, very time-consuming and costly sampling procedures and/or a heavy burden of equipment operation and maintenance. Thus, it is still challenging but necessary to develop a new method showing high selectivity, sensitivity, and rapid determination for routine measurement after naproxen administration.

A ligand binding assay (LBA) provides an indirect measure of the interactions between the analyte of interest and its specific targets in complex matrices [26,27]. Given that antibodies are the most common targets of endogenous or exogenous soluble ligands, certain classic immunoassays (ELISA, radioimmumoassay, magnetic immunoassay, fluorescence immunoassay, colloidal gold immunochromatographic assay, chemiluminescence immunoassay) have been widely applied in the analysis of biological macromolecules and small molecules. However, most of these immunoassays, especially those applied for small drug determination, require expensive antibodies and tedious and time-consuming preparation procedures. Due to the continuous investigation of the human serum albumin (HSA) binding properties of naproxen [28,29], a low-cost competitive protein binding assay (CPBA) with a binding rate of more than 99% was developed in this study. Our new method probably offers low cost, high resolution and high capacity for an analytic approach using HSA as a target to bind its partner, naproxen.

Using nanomaterials as carriers for the recognition of antibodies to obtain signal amplification is an emerging and promising direction for immunoassays. It was reported that a large number of nanoparticles were adopted as solid carriers to improve the performance of conventional ELISA, as a result of the strong absorption ability and high surface-to-volume ratio of the nanoparticles [30]. The miniaturized nanoscale material lends itself to a small diffusion distance compared with traditional plate wells, and thereby promotes faster reagent diffusion times [31]. Silicon dioxide nanoparticles (nano-SiO_2_) represent a type of important nanomaterial with a wide range of applications, and are characterized by easy preparation, large surfaces areas, high chemical stability, and good biocompatibility [32]. In the present study, a nano-SiO_2_ based CPBA method was developed for naproxen determination using nano-SiO_2_ as a carrier, HSA as a binding receptor and horseradish peroxidase (HRP) as a marker enzyme. Using the proposed method, a nano-SiO_2_ based competitive protein binding assay (nano-CPBA) was performed for naproxen measurement in a human urine sample with the advantages of low cost, fast speed, high accuracy, and simple equipment. Our method would provide more flexibility to expand the routine determination of small molecular drugs in biological samples.

## 2. Results and Discussion

**Figure 1:**
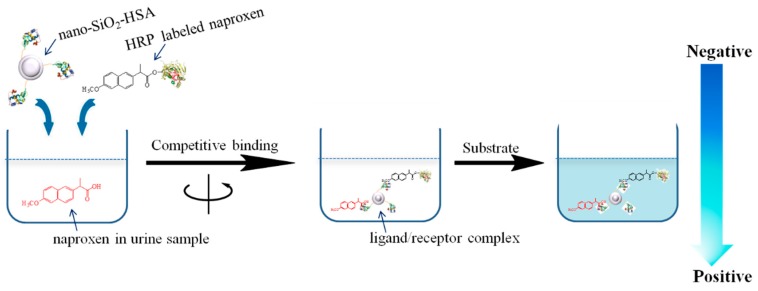
Illustration of naproxen determination by nano-competitive protein binding assay (CPBA).

The present work describes an enzyme-linked biorecognition analytical method based on ligand (naproxen)-receptor (HSA) binding. It is well known that naproxen is capable of binding to HSA in the subdomain IIIA site with high affinity. As illustrated in Figure 1, free naproxen in the sample and HRP-labeled naproxen (HRP-naproxen) competitively bind to HSA which was immobilized on the surface of silicon dioxide nanoparticles. Some of the binding sites on HSA binding to free naproxen were measured, while others binding to HRP-naproxen. After centrifugal separation of excessive HRP, the catalytic color reactions were investigated on an HRP/TMB/H_2_O_2_ system [33,34,35] by the HRP-naproxen/nano-SiO_2_-HSA composite. The free naproxen to be measured and HRP-naproxen added were competitively bound to HSA, thus the higher content of naproxen in the sample, the less HRP-naproxen binding to HSA, resulting in a weaker color of the substrate solution. Although the idea was referred to in our previous work on ibuprofen [35], we studied naproxen in this work to confirm that competitive protein binding assay can be used for routine determination of small molecular drugs in biological samples. It is a demonstration of the utility of this sensing strategy.

### 2.1. Characterization

**Figure 2:**
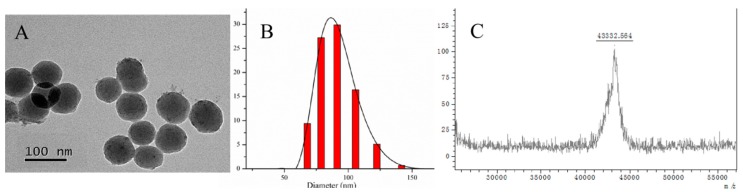
The TEM image (**A**) and size distribution (**B**) of human serum albumin functionalized silicon dioxide nanoparticles(nano-SiO_2_-HSA), and the molecular mass of horseradish-peroxidase-labeled naproxen (HRP-naproxen) measured by MALDI/TOF MS (**C**).

The main diameter of the particles was about 90 nm in accordance with the results shown by TEM (Figure 2A). The particle size distribution of nano-SiO_2_-HSA was measured by Malvern laser particle size analyzers, as shown in Figure 2B. The coupling ratio of HRP-naproxen was calculated to be about 3:1 which was determined by MALDI/TOF MS as shown in Figure 2C.

### 2.2. Optimization of HRP-Naproxen Dilution

**Figure 3:**
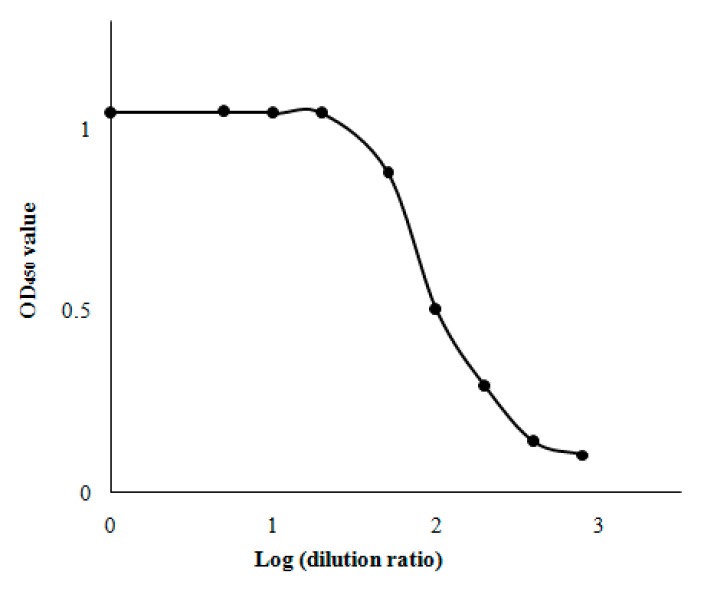
Optimization of HRP-naproxen dilution by the OD_450_ values versus the logarithm of HRP-naproxen.

As a direct competitive method, for optimization of the ratio of SiO_2_-HSA/HRP-naproxen, there needs to be sufficient HRP-naproxen to combine with SiO_2_-HSA, given that the nanoparticles should bind to all the HRP-naproxen when the system does not contain any naproxen. As shown in Figure 3 the OD_450_ values (y) versus the logarithm of HRP-naproxen dilution from 1:1 to 1:800 (x). A clear turning point at (*x* 1.30, *y* 1.04) could be observed from the graph, corresponding to a dilution of 1:20. Thus, the dilution of1:20 was set as an optimal parameter for the nano-CPBA analysis.

### 2.3. Analytical Figures of Merit

The calibration curve was established as y = −0.006x + 1.023 with good correlation (r^2^ = 0.997) based on linear regression analysis of the ratio of OD_450_ values between standard solutions (B) and blank (B_0_) (y, B/B_0_) versus standard (x, ng/mL) at six different concentrations ranging from 5 ng/mL to 150 ng/mL. The limit of detection (LOD) was determined as 2.36 ng/mL. The precision of the present nano-CPBA method was evaluated by the coefficient of variation (CV) of intra-day and inter-day assays, which were 3.7% and 5.1%, respectively. The average content of sample 2 (sampling time-point at 2.5 h after a single oral dose of 100 mg of naproxen tablet) by six independent determinations was 151.79 ± 3.08 ng/mL, which indicated that the reported analytical method exhibited high repeatability. The recovery was evaluated by spiking naproxen standard of high, middle and low amounts (180 ng/mL, 150 ng/mL and 120 ng/mL) to sample 2, respectively. Another recovery was evaluated by spiking naproxen standard of 120 ng/mL to untreated urine sample as received by the volunteers. As shown in Table 1, the CVs ranged from 4.49% to 8.29%, while the mean recovery ranged from 89.94% to 97.98%, which showed an ideal recovery rate. Thus, the results indicate that the reported nano-ELISA method could be applied to naproxen determination in urine.

### 2.4. Determination of Naproxen in Urine by Nano-CPBA Method

**Figure 4:**
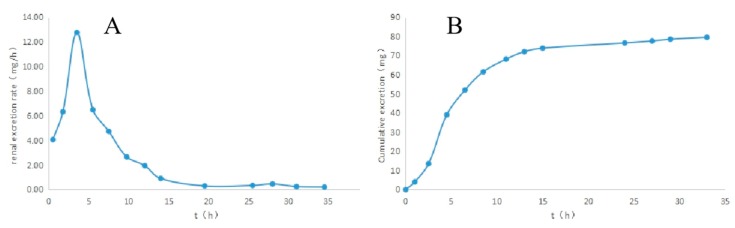
The urinary excretion rate-time curve of naproxen (**A**) and urinary cumulative excretion-time curve of naproxen (**B**).

Following the procedures described above, the newly developed method was applied to the continuous measurement of naproxen in urine donated by healthy volunteers. The validation of this method and a recovery test were carried out on spiked samples of urine. A total of 13 urine samples were collected within 36 h of a single oral administration of naproxen tablet to each human subject. We determined the amount of naproxen in human urine using this nano-CPBA method. Some samples outside of the range of the calibration curve were diluted by blank urine to place them within the range. The amount of naproxen detected at levels ranging from 9.15 ± 3.07 to 408.40 ± 11.25 ng/mL in the interval from 0–36 h showed that 85.5% of the dose was excreted in the urine over 36 h, which was consistent with recovery of 83% of the dose reported in normal adult volunteer subjects [36]. As shown in Figure 4A, during the period of 2.5–4.5 h, the renal excretion rate peaked and then decreased rapidly. Figure 4B showed that an apparent urinary excretion of naproxen was found within 1 h after oral administration, and 92.1% of the total recovered drug in urine was excreted in 0-15 h after oral administration.

## 3. Materials and Methods

### 3.1. Chemicals and Materials

Naproxen standard (CAS 22204-53-1), 1-(3-dimethylaminopropyl)-3-ethylcarbodiimide hydrochloride (EDC.HCl), N-hydroxysuccinimide (NHS), 3-aminopropyltrimethoxysilane (APTMS), horseradish peroxidase (HRP) and 3,3′5,5′-tetramethylbenzidine (TMB) were purchased from Shanghai Titan Chemical Co., Ltd. (Shanghai, China). Tetraethylorthosilicate (TEOS), ethanol, ammonia solution (NH_3_.H_2_O), hydrogen peroxide (H_2_O_2_) and other chemicals of AR grade were purchased from Sinopharm Chemical Reagent Co., Ltd. (Shanghai, China). Human serum albumin (HSA) was purchased from Shanghai Xinyu Biological Technology Co., Ltd. (Shanghai, China). Naproxen tablets (batch number 151203) were purchased from Nanjing BaiJingYu Pharmaceutical Co., Ltd. (Nanjing, China). Urine samples were donated by volunteers.

### 3.2. Instrumentation

The UV absorbance was measured with a UV-L5S ultraviolet spectrophotometer (Shanghai INESA Scientific Instrument Co., Ltd., Shanghai, China). The sizes and morphologies of nano-SiO_2_ were observed with a FEI Tecnai G20 transmission electron microscope (FEI, Co., Hillsboro, OR, USA). The particles size distribution of nano-SiO_2_-HSA was measured with a laser particle size analyzer (Zetasizer Nano, the Malvern Zetasizer, Malvern, UK). The thermo-gravimetric analysis was obtained by heating powdered samples from room temperature to 800 °C under nitrogen atmosphere using a TGA Q500 V20.13 Build 39 thermo-analysis system (TA, Co, New Castle, DE, USA). MALDI-TOF mass data was obtained on a Bruker Autoflex TOF mass spectrometer (Bruker Daltonic, Billerica, MA, USA) operated in linear high internal mode.

### 3.3. Preparation of HSA Immobilized Nano-SiO_2_ Nanoparticles (SiO_2_-HSA)

The HSA immobilized nano-SiO_2_ nanoparticles (SiO_2_-HSA) were prepared following our similar procedures reported previously [33,34,35]. Briefly, SiO_2_ nanoparticles (nano-SiO_2_) were firstly prepared by the hydrolysis of TEOS according to the typical Stöber process. Secondly, the particles were dispersed in APTMS solution to obtain amine-terminated SiO_2_ nanoparticles (SiO_2_-NH_2_). Thirdly, the SiO_2_-HSA nanoparticles were prepared by the carbodiimide crosslinking method using the SiO_2_-NH_2_ and HSA. About 50 mg HSA dispersed in 40 mL PBS solution (10 mM, pH 7.4) was added dropwise into a 1 g SiO_2_-NH_2_ suspension dispersed in 10 mL PBS solution with constant stirring. Then, 2 mL solutions containing 400 mg/mL of EDC and 250 mg/mL NHS were added into the above mixture. After reaction for 12 h, the solution was washed three times with deionized water with centrifugation separation. The SiO_2_-HSA nanoparticles were obtained, dispersed in 100 mL PBS and stored at 4 °C for the further use.

### 3.4. Preparation of HRP Labeled Naproxen (HRP-Naproxen)

HRP-labeled naproxen (HRP-naproxen) was prepared by the active ester method for its high efficiency and convenient operation: 46 mg N,N-dicyclohexylcarbodiimide and 44 mg sulfo-NHS were successively added into 8 mL N,N-dimethylformamide containing 46 mg naproxen with overnight stirring in an ice bath. After centrifugal separation at 4000 rpm for 5 min, the supernatant was dropped into 20 mL HRP solution (1 mg/mL, PBS) and reacted for 8 h. After dialyzing for 24 h at 4 °C, HRP-naproxen was obtained and stored at 4 °C for further use.

### 3.5. Optimization of HRP-Naproxen Dilution

As a direct competitive method, optimization of the ratio of SiO_2_-HSA/HRP-naproxen needs to ensure that there is enough HRP-naproxen to combine with SiO_2_-HSA, given that the nanoparticles would bind to all the HRP-naproxen when the system does not contain any naproxen. Briefly, a total of 9 batches of 0.5 mL SiO_2_-HSA suspension liquid, diluted by with 0.5 mL PBS solution (10 mM, pH 7.4), mixed with 0.5 mL HRP-naproxen solution with a series of gradient dilution (1:1, 1:5, 1:10, 1:20, 1:50, 1:100, 1:200, 1:400, and 1:800, respectively), were incubated at 20 °C for 15 minutes. After being washed three times with 1 mL PBS solution (10 mM, pH 7.4) coupled with centrifugal separation, the precipitation was incubated with 1 mL of TMB substrate solution (2 mg TMB and 0.1 mL H_2_O_2_ dissolved in 20 mL of 0.1 M citrate-phosphate buffer) for 15 min at 20 °C. After termination of the reaction with 2 M H_2_SO_4_, the OD_450_ values of the supernatant were measured with a UV spectrophotometer.

### 3.6. Urine Samples Analysis

The newly developed nano-CPBA method was applied to the continuous test of naproxen in urine donated by healthy volunteers with a single oral dose of a 100 mg naproxen tablet. The principle of the direct competitive nano-CPBA method was the same as that of a classic direct competitive ELISA. As illustrated in Figure 1: first, 0.5 mL of the urine sample and 0.5 mL of the suspension liquid of SiO_2_-HSA nanoparticles were mixed together and incubated for 15 min at 37 °C. Subsequently, 0.5 mL of HRP-naproxen solutions was added into the above incubation system for 15 min. After being washed with PBS solution three times coupled with centrifugal separation, the precipitation was incubated with 1 mL of TMB substrate solution (2 mg TMB and 0.1 mL H_2_O_2_ dissolved in 20 mL of 0.1 M citrate-phosphate buffer) for 15 min at 20 °C, then terminated with 2 M H_2_SO_4_. Finally, the OD_450_ values of the supernatant were measured for calculation with a UV spectrophotometer.

### 3.7. Method Validation

A 10 mg/mL stock solution of naproxen standard was prepared, with N,N-dimethylformamide as a solvent. Then, six work solutions I–VI were diluted with PBS solution to the necessary concentrations (5 ng/mL, 10 ng/mL, 20 ng/mL, 50 ng/mL, 100 ng/mL and 150 ng/mL, respectively). The calibration graph was plotted based on binary linear regression analysis using a ration of OD_450_ values (y, B/B_0_) versus concentrations (x, ng/mL) of different standard concentrations, where B was the OD_450_ values of six work solutions I–VI deducting the blank value, and B_0_ was PBS solution. The LOD was defined as the result of the average value minus three times the standard deviation value. The precision was investigated by the intra-day and inter-day variations which were determined by a real urine sample solution (sample 2, sampling time-point at 2.5 h after a single oral dose of a 100 mg naproxen tablet) within 12 h and within three days, respectively. The repeatability was investigated by six independent determinations of sample 2. Recovery was determined by spiking three different amounts of naproxen standard (120%, 100% and 80% of naproxen content in the sample) to sample 2, and then analyzed according to the aforementioned nano-CPBA procedures.

## 4. Conclusions

In this work, a simple, direct and low-cost analytical method was developed for the determination of naproxen based on a nano-SiO_2_ competitive protein binding assay (nano-CPBA). The results of the experiments demonstrated that the introduction of HSA in place of the pharmacological receptor in a conventional immunoassay significantly reduced the test costing, expanded the coverage and provided more flexibility in a routine test on a biological sample. Nano-SiO_2_, as a stationary phase with a high surface-area-to-volume ratio, markedly enhanced the substantial adsorption of ligand (analyte) and receptor (HSA) interaction in determining naproxen. This was contributed to the improvement of analytical performance, which showed a good linear relationship, shortened testing time and enhanced repeatability and reproducibility. As a proof of principle, this novel assay was successfully validated to be suitable for the quantification of naproxen in urine with the advantages of high sensitivity and accuracy, while also having a relatively low cost and instrument demand. The method described in this paper is capable of expanding the analytical applications of nanomaterials and of determining other small-molecule compounds from various biological samples.

## Figures and Tables

**Table 1:** Results of the recovery evaluation at three spike amount levels with treated and untreated urine samples.

Naproxen Content in Urine Samples (ng/mL)	Added (ng/mL)	Detected (ng/mL)	Recovery (%)	Mean Recovery (%)	SD	CV (%)
152	180	302	83.4	89.9	0.07	8.29
152	180	311	88.4			
152	180	328	98.0			
152	150	285	88.9	95.8	0.06	6.22
152	150	301	99.5			
152	150	300	99.0			
152	120	275	102.9	98.0	0.04	4.49
152	120	265	94.4			
152	120	268	96.6			
0	120	117	97.2	93.5	0.02	4.11
0	120	107	89.5			
0	120	112	93.6			
